# Chloroquine as a promising adjuvant therapy for type 1 *Diabetes Mellitus*

**DOI:** 10.1038/s41598-020-69001-2

**Published:** 2020-07-21

**Authors:** Renato Ferreira de Almeida Júnior, Karla Simone Costa de Souza, Ony Araujo Galdino, Arnóbio Antônio da Silva Junior, Ricardo Fernando Arrais, Paula Renata Lima Machado, Kleber Juvenal Silva Farias, Adriana Augusto de Rezende

**Affiliations:** 1 Department of Clinical and Toxicological Analyses, Federal University of Rio Grande Do Norte (UFRN), Av. General Gustavo Cordeiro de Farias, S/N, Faculdade de Farmácia, Petrópolis, Natal, RN CEP: 59012-570 Brazil; 2 Department of Pediatrics, Pediatric Endocrinology Unit, Federal University of Rio Grande Do Norte, (UFRN), Natal, RN 59012-570 Brazil; 3 Department of Pharmacy, Federal University of Rio Grande Do Norte (UFRN), Natal, RN 59012-570 Brazil; 4 Center of Education and Health, Federal University of Campina Grande, Campina Grande, PB 58175-000 Brazil

**Keywords:** Cytokines, Diabetes complications, Type 1 diabetes

## Abstract

Chloroquine (CQ) and hydroxychloroquine, are promising anti-inflammatory drugs for the treatment of *Diabetes mellitus* (DM) to prevent associated complications. Therefore, this study evaluated the anti-inflammatory effects of CQ-free and CQ-incorporated polylactic acid nanoparticles (NPs) in the peripheral blood mononuclear cells (PBMCs) of patients with type 1 *Diabetes mellitus* (T1DM). In total, 25 normoglycemic individuals and 25 patients with T1DM aged 10–16 years were selected and glycemic controls evaluated. After cell viability assessed by MTT assay, T1DM PBMCs were subjected to a CQ concentration of 10 µM in three different conditions: not treated (NT), treated with CQ, and treated with CQ NPs. The cells were incubated for 48 h, and the mRNA expressions of cytokines *IL1B, IFNG, TNFA, IL12,* and *IL10* were determined by relative quantification through real-time PCR at 24 h intervals. *IL1B* expression decreased in CQ and CQ NP-treated cells after 48 h (p < 0.001) and 24 h (p < 0.05) of treatment, respectively. *IFNG* and *IL12* expressions significantly decreased (p < 0.001) in cells treated with CQ and CQ NPs at 24 and 48 h compared to NT. *TNFA* and *IL10* expressions significantly decreased after 48 h (p < 0.001) and 24 h (p < 0.002), respectively, by both CQ and CQ NPs treatment. Despite being a preliminary in vitro study, CQ has anti-inflammatory activity in the primary cells of T1DM patients and could represent an alternative and adjuvant anti-inflammatory therapy to prevent diabetes complications.

## Introduction

*Diabetes mellitus* (DM) is a serious health concern worldwide and patients with diabetes develop health problems due to complications, leading to the need for increased medical attention, a reduced quality of life, and premature death^1,2^. According to the International Diabetes Federation, more than 463 million people had diabetes in 2019, which is projected to reach 700 million in 2045. Therefore, continuous medical monitoring is necessary to reduce the risk of complications^2^.

Type 1 DM (T1DM) occurs primarily in childhood with over 100,000 children and adolescents diagnosed each year. It is a chronic autoimmune disease characterized by hyperglycemia due to the destruction of pancreatic β cells, leading to an absolute deficiency of insulin^1,3^. In the long run, inadequate glycemic control leads to the formation of advanced glycation end products (AGEs), which in turn cause changes to synthesis and damage to tissue structure and function. In addition, AGEs activate the signaling cascade that causes an intense inflammatory response with the production of reactive oxygen species in nerve and endothelial cells, causing microvascular (nephropathy, neuropathy, and retinopathy) and macrovascular (coronary disease, cerebrovascular disease, and peripheral arterial disease) lesions^4,5^.

Generally speaking, this event occurs when damage-associated molecular pattern proteins, AGEs, high mobility group box-1 proteins, heat shock proteins, and growth-specific protein 6 bind to toll-like receptors (TLRs) 2 and 4 on the β-cell membrane. Then, the activation of nuclear factor *κB* (NF-κB) occurs and, consequently, the transcription of pro-inflammatory cytokines, such as IL-1α, IL-1β, IL-8, IL-10, IL-12, and IFN-α, which, with the recruitment of CD4 + and CD8 + T cells and macrophages, leads to an intense inflammatory infiltrate in the pancreatic islets^6,7^.

Currently, the control of the inflammatory response occurs through the stabilization of glycemia, which is achieved by using insulin as a monotherapy^8^. Although this therapy is beneficial, the use of an adjunctive anti-inflammatory therapy would reduce the immune response and consequently delay the complications of diabetes. In this context, drugs acting on the immune system could play a role in preventing and treating these complications^9,10^. One of these drugs with promising prospects is chloroquine (CQ)^11,12^.

CQ is a 9-aminoquinoline first discovered in 1934 and used in the early twentieth century as the drug of choice against malaria, and routinely used as an anti-inflammatory agent to treat chronic diseases, such as systemic lupus erythematosus (SLE) and rheumatoid arthritis (RA)^13^. In addition to its use in treating chronic diseases, CQ is being studied both in vitro and in vivo as an anticancer therapy, and has subsequently displayed tumor toxicity and improved autophagy and immune system responses^14,15^. CQ is a safe drug with low toxicity, which when administered acutely or chronically has few adverse effects^16^.

Previous studies have indicated that CQ acts by modulating the immune system, primarily by blocking antigen presentation and thereby preventing the activation of the innate and acquired immunogenic systems responsible for the production of inflammatory cytokines. Therefore, the use of CQ in SLE and RA reduces acute and chronic inflammation and lessens the systemic complications caused by these diseases^11,17,18^. Although the mechanism by which CQ acts to modulate the immune response remains uncertain, it has been reported that the accumulation of CQ within lysosomes, lymphocytes, and macrophages generates pH increases, thereby altering the processing and assembly of major histocompatibility complex (MHC) II^19–22^. Other studies have proposed that CQ binds to autoantibodies within endosomes, preventing them from binding to endosomal TLRs 3, 7, and 9, and consequently blocking the expression of proinflammatory cytokines by the activation of *NF-κB*^22,23^.

Therefore, because the mechanism of action of CQ occurs in the cytosol of inflammatory cells, CQ has been incorporated into polylactic acid (PLA) nanoparticles (NPs)^24,25^, since these NPs have electronic affinity with the plasma membrane, which facilitates the endocytosis of the drug^26–28^. In addition, PLA is a polymer that is approved by the Food and Drug Administration (FDA) and widely used in the encapsulation of various drugs and has immunostimulatory capacity and immunosuppressive regulation, thereby increasing the presentation of antigen-presenting cells and blocking CD8 + ^28,29^.

Considering the inflammatory characteristics of T1DM and the anti-inflammatory properties of CQ, this study, initially in vitro*,* was conducted to investigate the potential of CQ as an adjunctive anti-inflammatory therapy. This was achieved by evaluating the mRNA expressions of cytokines *IL1B, IFNG, TNFA, IL12*, and *IL10* in patients’ peripheral blood mononuclear cells (PBMCs) treated with CQ-free formulations and incorporated into PLA NPs.

## Results

### Biochemical analysis

The characteristics of the study population demonstrate that there was no statistical difference regarding the age of the patients (normoglycemic group (NG) = 12.3, T1DM = 14.4, p = 0.065), indicating the homogeneity of the individuals. For patients with T1DM, the age at diagnosis was, on average, 9 years of age, and the time to diagnosis was 4 years.

For glycemic data, a statistical difference was found between patients with T1DM and NG individuals (T1DM = 194 mg/dL and 9.9% and NG = 96 mg/dL and 4.9% of fasting glucose and glycated hemoglobin (HbA1C), respectively, p < 0.05). This demonstrated that T1DM patients have values above those that are recommended by the American Diabetes Association (ADA) for good glycemic control^1^.

### PBMC viability after treatment with CQ and CQ NPs

**Figure 1:**
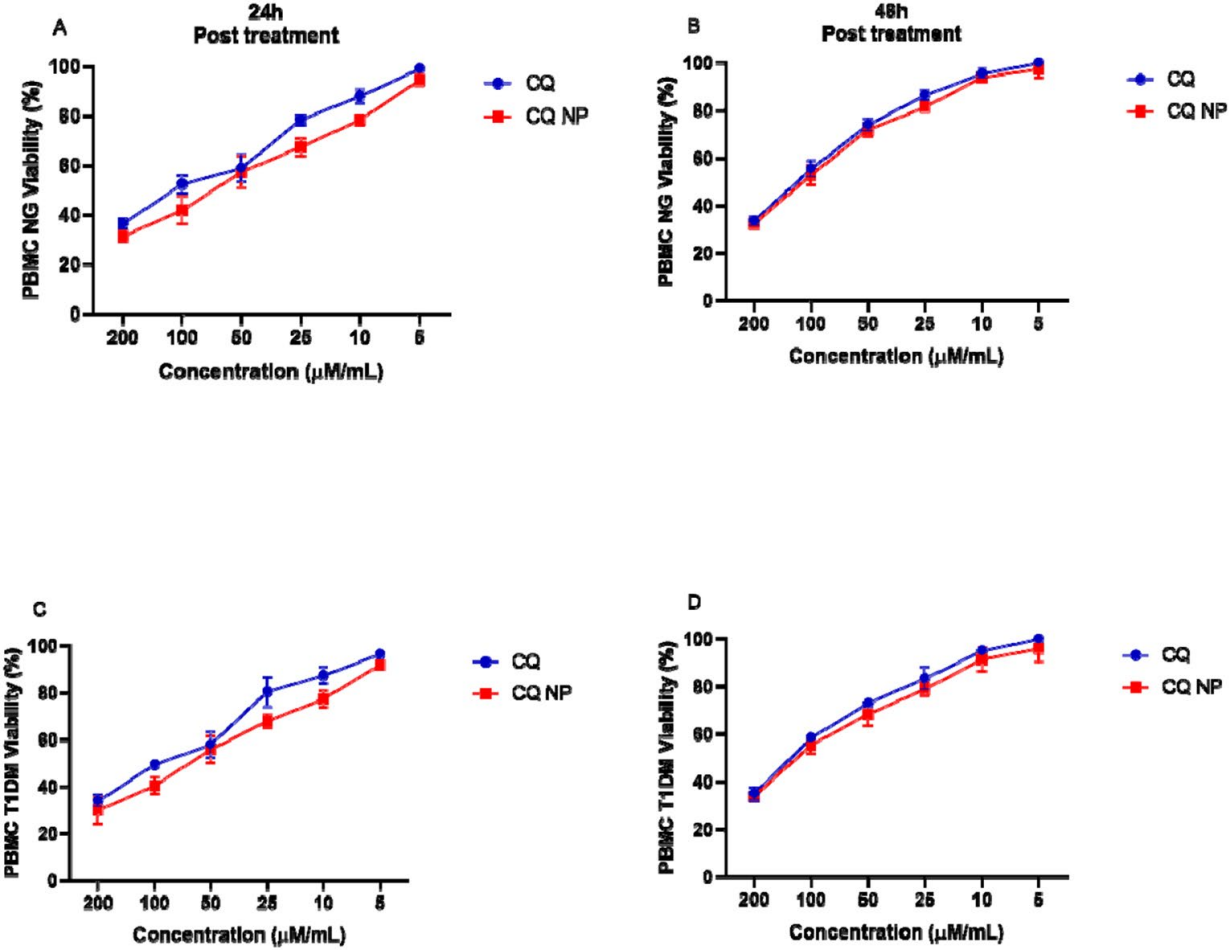
Cell viability assessed using the MTT method in the PBMCs of NG and patients with T1DM exposed to different concentrations of CQ and CQ NPs. The cytotoxic effect of CQ and CQ NPs in the PBMCs of NG individuals after incubation for 24 and 48 h (**A**,**B**). Cytotoxicity of CQ and CQ NPs in the PBMCs of patients with T1DM after incubation for 24 and 48 h (**C**,**D**).

PBMC viability after treatment with CQ and CQ NPs is shown in Fig. 1. There was no significant difference in the viability of PBMCs of the NG and T1DM groups treated with CQ or CQ NPs after 24 (Fig. 1A,C) and 48 h (Fig. 1B,D) of treatment. CQ concentrations greater than 25 μM had low cell viability and those below 10 μM presented viability close to 100%. The concentration of 10 μM was selected for the in vitro study of CQ immunomodulatory action. In this study, it was found that the mean 50% cytotoxic concentrations (CC50s) for the PBMCs of NG individuals and patients with T1DM were 97.2 µM for CQ and 70.3 µM for CQ NPs after 24 h of treatment, and 124.37 µM for CQ and 108.33 µM for CQ NPs after 48 h.

### The effects of CQ and CQ NPs on the expression of PBMC cytokines *IL1B, INFG, TNFA, IL12*, and *IL10*

**Figure 2:**
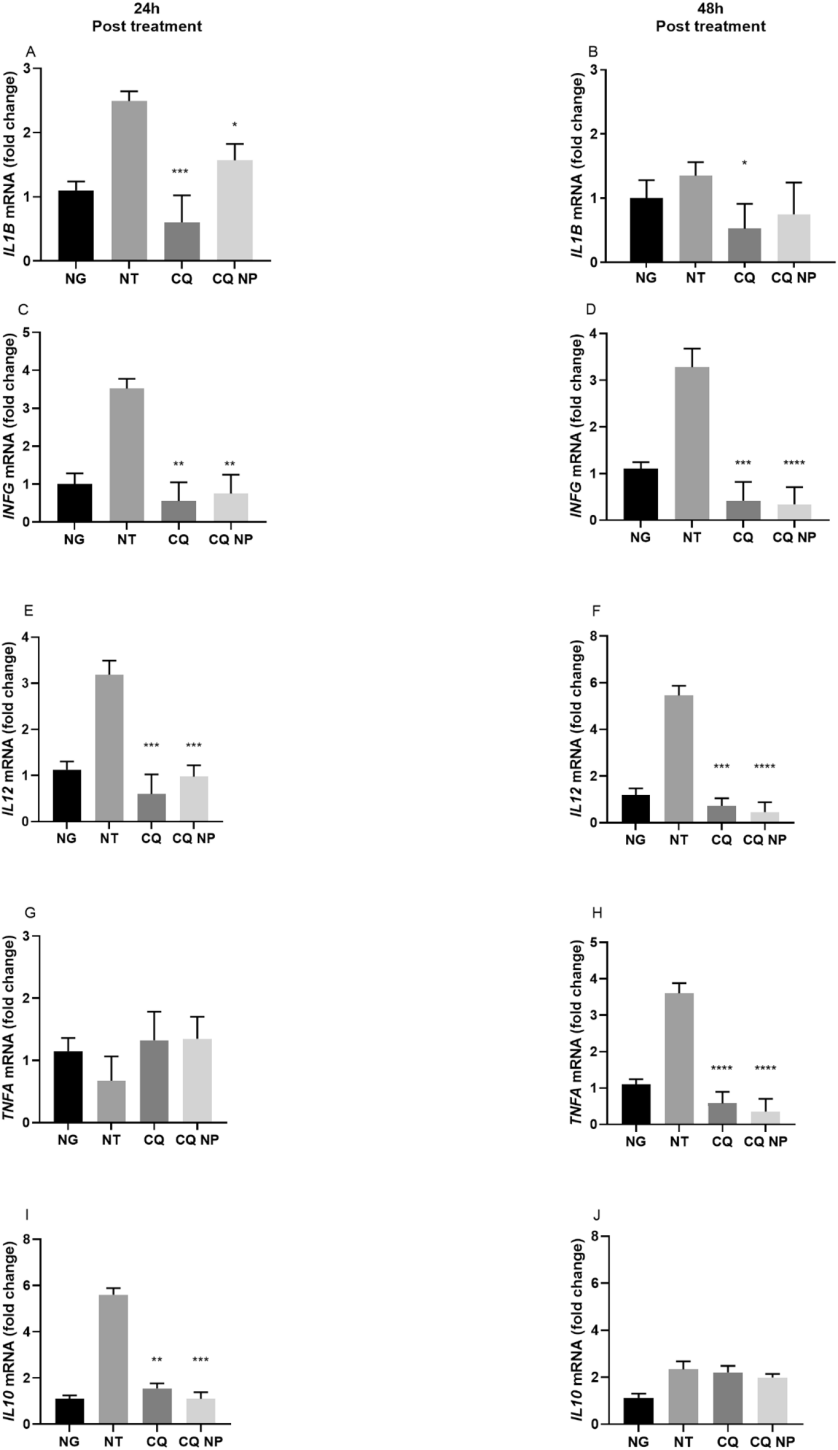
The cytokine gene expression in PBMCs of NG individuals; T1DM untreated (NT); patients treated with CQ (CQ); patients treated with CQ NPs (CQ NP) in 24 h with *IL1B* (**A**), *INFG* (**C**), *TNFA* (**E**), *IL12* (**G**), and *IL10* (**I**) and up to 48 h with *IL1B* (**B**), *INFG* (**D**), *TNFA* (**F**), *IL12* (**H**), and *IL10* (**J**). The results are presented as double changes in the values of NG media, normalized to *ACTB*, and expressed by the mean ± SEM. Significance compared to PBMCs of patients with T1DM undergoing treatment ****p < 0.001, ***p < 0.002, **p < 0.003, *p < 0.05. Analysis was performed with ANOVA followed by Tukey’s multiple comparisons test.

The evaluation of gene expression of the cytokines in PBMCs of patients with T1DM treated with CQ and CQ NPs is shown in Fig. 2. *IL1B* expression decreased in CQ-treated cells for up to 48 h of treatment compared to cells of no treatment (NT) patients (p < 0.001), whereas for CQ NP cells, this result was observed within only 24 h of treatment (p < 0.05) (Fig. 2A,B). *IFNG* and *IL12* gene expressions significantly decreased (p < 0.001) in cells treated with CQ and CQ NPs at 24 and 48 h compared to NT (Fig. 2C–F). *TNFA* later significantly decreased, and cells treated with CQ and CQ NPs experienced these decreases after only 48 h of treatment (p < 0.001) (Fig. 2G,H). *IL10* expression only showed significant reductions during 24 h of CQ and CQ NP treatment (p < 0.002) (Fig. 2I,J).

## Discussion

This is a preliminary study evaluating the effect of CQ as an adjuvant anti-inflammatory treatment for T1DM. The results revealed that CQ reduced the gene expression of inflammatory cytokines, indicating its potential in the control and reduction of complications associated with diabetes. Importantly, the CQ NP formulation also represents a promising innovation as a vehicle of CQ for its intracellular action and improved control in prolonged treatments.

Currently T1DM treatment is monotherapeutic through the use of insulin; however, glycemic control alone is unable to improve the immune response^30^. Therefore, adjuvant use of an anti-inflammatory, such as CQ, would improve the immune response and prevent the progression of complications. Although several studies have demonstrated the adjuvant action of anti-inflammatory drugs in controlling inflammation in diabetes, such as metformin, sulfonylureas, peptide 1 agonists, and a glucagon-like receptor, dipeptidyl peptidase 4^31^, it is important to note that these studies were carried out for type 2 diabetes ^30,32^. As a result, CQ could be an alternative for patients with diabetes, particularly those with T1DM.

CQ has good pharmacokinetic parameters, such as high solubility in aqueous media, good absorption by the gastrointestinal tract, and bioavailability between 50 and 74%. However, CQ exhibits toxicity after prolonged use due to its high half-life^18^. This problem could be reduced through the use of nanotechnology, and in the present study CQ was incorporated into PLA NPs to decrease toxicity, improve the entry of the drug into cells, and facilitate its excretion^33^.

Therefore, we first assessed the viability of PBMCs against CQ and PLA NPs. We observed that only high concentrations of CQ or CQ NPs were toxic, and that concentrations below 25 µM were viable above 80%. We also found that CC50 was almost 10 times greater than the concentration used in the study (10 µM), demonstrating the safety of the drug. The use of this concentration corroborates the research carried out by Schmidt et al. who found that this concentration was also beneficial in anti-inflammatory therapy in patients with RA^34^. In addition to the data obtained in this assay, in which the CC50 of the CQ NPs was higher than that of the free CQ, we suggest that the incorporation of the drug in PLA NPs facilitates the entry of CQ into cells due to the differences in electrical charge in relation to the cell membrane.

In the inflammatory process, hyperglycemia generates end products of advanced glycation, which in turn implies the production and release of pro-inflammatory cytokines and free radicals and an increase in inflammation and oxidative stress, culminating in severe vascular complications^37–39^. Therefore, the potential anti-inflammatory effect of CQ observed by the reduced expression of cytokines in the PBMCs of patients with T1DM indicates that it is a good alternative to prevent vascular complications. As CQ increases the pH of endosomes, it could also prevent the activation of TLRs by altering the processing and assembly of MHC complex II, with consequent inhibition of inflammatory cytokines^19–22^.

These cytokines (IL-1β, IL-12, INF-γ, and TNF-α) can recruit inflammatory cells, such as Natural Killer (NK) and Dendritic cells (DC), in addition to promoting the maturation and stimulation of CD8 + and CD4 + T cells and plasmocytes^35,36^. They also promote the production of other inflammatory cytokines, such as IL-1α, IL-4, IL-6, IFN-β, and TNF-β, and the expression of apoptotic genes with Fas and Fas ligand, thus, causing severe tissue damage^36^.

These results are in accordance with those obtained by Jang et al., who demonstrated that CQ interferes in the synthesis of IL-1β, potentially through the processing of primary transcripts in the nucleus and transport of processed mRNA to the cytosol, causing mRNA degradation^17^. In addition, Stephen et al. observed that concentrations of 10 and 100 μM reduced the expression of *TNFA* mRNA in PBMCs, potentially in the pre-transcriptional stage, regardless of its trophic effect of lysosomes^37^.

For *IL10*, an anti-inflammatory cytokine that assists in modulating the immune response^38^, we observed that over 24 h and in a highly inflamed environment, the NT group showed higher *IL10* expression, as expected, compared to CQ and CQ NP-treated cells that show similar values to the NG individuals cells. The same results were observed over 48 h for CQ and CQNPs cells, most likely due to the inflammation already being controlled. However, a reduction of approximately three times was observed for NT cells, potentially due to the increase in Th1 standard cytokines, such as TNF-α and IL-12. A similar result was observed in a study carried out by Jiang et al. in patients with obstructive apneal syndrome^39^.

Limitations of this study include the cell model as primary cells of patients with T1DM have a short useful life, making it difficult to observe the anti-inflammatory and cytotoxic effects of CQ over long periods of time. However, the success of the study with this cell model in demonstrating the CQ anti-inflammatory effect for the first time, to the best of our knowledge, represents an important advance in the treatment of T1DM.

In conclusion, the reduced gene expression of inflammatory cytokines by CQ and CQ NPs in the PBMCs of patients with T1DM may represent an alternative adjuvant anti-inflammatory therapy that could prevent the vascular complications associated with T1DM. In addition, the CQ NP formulation also showed effective results by improving the entry of the drug into cells and presenting safe levels of toxicity for prolonged treatment.

Despite being an initial study carried out in vitro, it is possible to conclude that CQ has anti-inflammatory activity in the primary cells of patients with T1DM. Therefore, this study provides a new perspective for T1DM treatment and pre-clinical studies to confirm the anti-inflammatory effects of CQ as well as the possible prevention of vascular damage and complications. In addition, it may be possible to improve and better understand the pharmacodynamics of PLA NPs.

## Methods

### Preparation of CQ NPs

CQ was obtained commercially (Sigma-Aldrich Co. cat # C1386, lote # 081M1611V) and the CQ incorporated into PLA NPs was prepared according to the methods of Lima et al.^40^.

Briefly, CQ NPs were prepared using the emulsification-solvent evaporation method. The procedure was performed by mixing 0.4 mL of 0.5 M aqueous NaHCO_3_ (pH = 8.4) containing 1.25% v/v with 10 mL of organic phase dichloromethane containing PLA (0.25% v/v) to obtain a CQ:PLA ratio of 1:5 v/v. Then, the vial was vortexed for three cycles of 10 s at 1 min intervals. After, the supernatant (NaHCO_3_ solution) was removed and 4 mL of the organic phase containing CQ and PLA was injected at 1.0 mL/min in the aqueous phase (16 mL) containing the surfactant, poloxamer 407 (0.75% v/v), under magnetic stirring. The phases were filtered, and emulsification was produced under shaking via Ultra-turrax equipment (IKA Labortechnik, Staufen, Germany) with evaporation overnight. The samples were stored at 8 °C and the size and zeta potential were measured after 24 h^41^.

### Study population

Overall, 25 patients with T1DM aged between 10 and 16 years (T1DM group) were recruited from the Pediatric Endocrinology Unit, Onofre Lopes Universitary Hospital of the Federal University of Rio Grande do Norte (UFRN), Natal, RN, Brazil. All patients with T1DM were on continuous insulin. T1DM diagnosis was in accordance with the ADA criteria with fasting blood glucose ≥ 126 mg/dL and HbA1C ≥ 6.5%^1,3^. Glycemic controls were evaluated by measuring HbA1c in whole blood as well as the serum glucose concentration. All tests were performed using Wiener kits, according to the manufacturer’s instructions, using the biochemical analyzer CMD-800 (Wiener Laboratories, Rosario, Argentina).

In addition, 25 unrelated subjects (normoglycemic group, NG) with no previous diagnosis of T1DM, a fasting serum glucose of ≤ 99 mg/dL, and in the same age range were recruited from local public schools in Natal, RN, Brazil. The study was conducted in accordance with the guidelines set by the Ethics in Research Committee of the Federal University of Rio Grande do Norte, which complies with the Declaration of Helsinki, being approved by Research Ethics Committee of the Onofre Lopes Hospital of the UFRN under protocol number 704.310. Written informed consent was obtained from all adult subjects and parents or legal guardians of underaged patients and normoglycemic individuals. The exclusion criteria were pregnancy, hypertension, the presence of inflammatory diseases, and recent infections. After assessing medical history, fasting blood samples were obtained from all subjects for biochemical analyses, cell isolation, cell culturing, and extraction of total RNA.

### Isolation and culture of PBMCs

PBMCs were obtained from the NG and T1DM groups by density gradient, in which blood was added to Histopaque^®^ (Sigma-Aldrich, St Louis, MO, USA; density gradient 1.077 g/mL) and centrifuged to 400 × *g* for 30 min. After centrifugation the cells were resuspended in 2 mL RPMI 1,640 medium supplemented with 0.29 g/L L-glutamine, 2 g/L sodium bicarbonate, 25 mM/L HEPES, 1% antibiotic–antimycotic, and 10% serum. The cells were counted in a Neubauer chamber and checked for viability using trypan blue dye. After counting, the cells were diluted to obtain the required amounts for the experiments.

### PBMC viability after treatment with CQ and CQ NPs

The viability of the PBMCs of the T1DM group was assessed by MTT (Sigma-Aldrich, St Louis, MO, USA). Cells were cultured in 96-well plates with 1 × 10^4^ cells/well, exposed to CQ and CQ NP concentrations of 5, 10, 25, 50, 100, and 200 μM, and maintained in a CO_2_ stove (5%) at 37 °C for 48 h. Then, 5 mg/mL MTT was added to each well and the plate was incubated in a CO_2_ oven (5%) at 37 °C for 4 h. After, 100 μL of 10% sodium dodecyl sulfate in 0.001 N hydrochloric acid was added for dissolution of the crystals. The plate was then read in a microplate spectrophotometer at 540 nm (Biotek^®^, Epoch model, Winooski, Vermont, USA). The experiment was carried out in triplicate. Cell viability was defined in comparison to untreated controls. The relative cell viability (%) in the sample-treated wells with respect to the control wells was estimated by Eq. 1:

$${\text{\% Cell Viability = }}\frac{{{\text{(Tested)}} \times {100}}}{{\text{(Control)}}}$$

In which tested and control are the absorbance of treated sample and control sample, respectively.

To determine the CC50 values, nonlinear regression of concentration–response curves was determined. The CC50 was defined as the concentration that reduced cell viability by 50% compared to the untreated controls.

### T1DM PBMC treatment with CQ and CQ NPs

T1DM PBMC suspensions (1 × 10^5^ cells/well) were seeded in 24-well flat-bottomed culture plates (Corning Incorporated, Corning, New York, USA). The cells were subjected to three different conditions; no treatment (NT), treatment with CQ (CQ), and treatment with CQ NPs (CQ NP). Cells of the NG group were treated with culture medium only. The plates were maintained in a CO_2_ incubator (5%) at 37 °C for 24 and 48 h. Cells were then resuspended and collected at the end of each incubation period, then centrifuged at 500×*g* for 3 min to obtain the cells, and the pellet was used to obtain total RNA.

### Real-time quantitative polymerase chain reaction (qPCR) analysis

Total RNA was extracted from the cell pellet obtained from the T1DM and NG groups using a Promega SV Total RNA Isolation System (Promega Corporation, Madison, WI, USA), according to the manufacturer's instructions. The concentration and purity of the total RNA were measured using a nanodrop spectrophotometer (NanoDrop ND-1000, Montchanin, DE, USA) at 260 nm.

The Integrity of the RNA was determined by agarose gel electrophoresis (2% agarose/3-[N-morpholino] propanesulfonic acid) using aliquots of total RNA (approximately 10 μL). The agarose gel was stained with GelRed™ (Uniscience, São Paulo, SP, Brazil), revealing the presence of two sharp bands at approximately 5 and 1.8 Kb that corresponded to ribosomal RNA 28S and 18S, respectively.

Expression levels of *IL1B*, *IL12*, *IFNG*, *TNFA*, and *IL10* were evaluated via real-time qPCR. The normalizing reference gene was beta-actin (ACTB), which showed the greatest stability among the glyceraldehyde-3-phosphate dehydrogenase, hypoxanthine phosphoribosyltransferase 1, and beta-2-microglobulin genes after analyses by GENORM™ (v.3.44) and NORMFINDER software^42^.

All gene expression analyses were performed in a 7,500 Fast Real-Time PCR System (Applied Biosystems, CA, USA). Reactions were carried out in duplicate using a SuperScript^®^ III Platinum^®^ SYBR^®^ Green One-Step qRT-PCR kit (Life Technologies Corporation, Carlsbad, CA, USA) with a total volume of 12 μL containing approximately 10 ng of total RNA. Real-time PCR cycling conditions were as follows: 55 °C for 30 min, 95 °C for 10 min, followed by 40 cycles of 95 °C for 15 s and 60 °C for 1 min.

Relative expression was calculated using the 2^−ΔΔCt^ method^43^. Significance was determined via the comparison of ΔCt values for each target gene, and results are presented as the fold‐change of the NG group mean values normalized to *ACTB*.

### Statistical analysis

All data were expressed as mean ± standard deviation. The distribution of the variables was analyzed using the Kolmogorov–Smirnov test. Variables with normal distributions were subjected to Student’s t-test and ANOVA analysis, followed by comparison with Tukey's test. Data were analyzed in GraphPad Prism v.6.0 (GraphPad Software, Inc., San Diego, CA, USA). Statistically significant results were obtained at p < 0.05.
